# Novel insights into IL-37: an anti-inflammatory cytokine with emerging roles in anti-cancer process

**DOI:** 10.3389/fimmu.2023.1278521

**Published:** 2023-10-20

**Authors:** Min Gu, Yuexinzi Jin, Xun Gao, Wenying Xia, Ting Xu, Shiyang Pan

**Affiliations:** ^1^ Department of Laboratory Medicine, the First Affiliated Hospital of Nanjing Medical University, Nanjing, China; ^2^ Branch of National Clinical Research Center for Laboratory Medicine, Nanjing, China; ^3^ Center of Clinical Laboratory Medicine, Zhongda Hospital, Southeast University, Nanjing, China

**Keywords:** IL-37, inflammation inhibition, immunomodulation, anti-tumor, immunotherapy

## Abstract

Interleukin-37 (IL-37) is a newly discovered member of IL-1 family. The cytokine was proved to have extensive protective effects in infectious diseases, allergic diseases, metabolic diseases, autoimmune diseases and tumors since its discovery. IL-37 was mainly produced by immune and some non-immune cells in response to inflammatory stimulus. The IL-37 precursors can convert into the mature forms after caspase-1 cleavage and activation intracellularly, and then bind to Smad-3 and transfer to the nucleus to inhibit the production and functions of proinflammatory cytokines; extracellularly, IL-37 binds to cell surface receptors to form IL-37/IL-18Rα/IL-1R8 complex to exert immunosuppressive function via inhibiting/activating multiple signal pathways. In addition, IL-37 can attenuate the pro-inflammatory effect of IL-18 through directly or forming an IL-37/IL-18BP/IL-18Rβ complex. Therefore, IL-37 has the ability to suppress innate and acquired immunity of the host, and effectively control inflammatory stimulation, which was considered as a new hallmark of cancer. Specifically, it is concluded that IL-37 can inhibit the growth and migration of tumor cells, prohibit angiogenesis and mediate the immunoregulation in tumor microenvironment, so as to exert effective anti-tumor effects. Importantly, latest studies also showed that IL-37 may be a novel therapeutic target for cancer monitoring. In this review, we summarize the immunoregulation roles and mechanisms of IL-37 in anti-tumor process, and discuss its progress so far and potential as tumor immunotherapy.

## 1 Introduction

Interleukin 37 (IL-37) is a recently identified cytokine belonging to the IL-1 superfamily. Based on the consensus sequence, shared receptor and the co-receptor, the IL-1 family cytokines are divided into three subgroups: including IL-1 subfamily (IL-1α, IL-1β, IL-1Ra and IL-33), IL-18 subfamily (IL-18 and IL-37), and IL-36 subfamily (IL-36α, IL-36β, IL-36γ, IL-36Ra and IL-38) (1). In recent years, increasing studies have focused on the key regulatory roles of IL-1 family cytokines in innate and adaptive immunity, for example, the modulation of lymphocyte differentiation and function in the pathophysiology of human cancers, among which the anti-inflammatory cytokine IL-37 is highlighted.

IL-37, formerly termed IL-1 family member 7 (IL-1F7), is a novel anti-inflammatory cytokine of the IL-1 family (1, 2). Since the first discovery of IL-37 in 2000 through in silico analysis from gene data banks by Kumar et al. (3), multiple studies have established that IL-37 was crucial in the coordination of diversity and plasticity of both innate and adaptive immune responses via downregulating the production and function of pro-inflammatory cytokines/chemokines (4–7), thereby served as a negative regulator in inflammatory diseases such as infectious disease, allergic disease, metabolic disease and autoimmune diseases, etc. (8–10). In contrast to other members of the IL-1 family, far less is currently understood concerning the role of IL-37 in cancer, it may be related to the fact that its murine or chimpanzee homologue has not yet been discovered (6). Recently, Hanahan D et al. considered that inflammation could be one of the hallmarks of tumors (11) and is inextricably associated with tumorigenesis (12–14). In addition, accumulated data suggested that the anomalous expression of IL-37 is involved in neoplasms progression and metastasis from different systems through various fashions, including regulating a series of cytokines and molecules in tumor-related signaling pathways (15–19), providing evidence that IL-37 was crucial in regulating the occurrence, pathogenesis and prognosis of human cancers. In this review, we draw on recent advances to provide update on the biological characteristics of IL-37 and its clinical potential as immunological regulator, therapeutic targets as well as disease modifiers in cancer.

## 2 Basic characteristics

### 2.1 Structure and isoforms of IL-37

*IL-37* gene is located on the chromosome band 2q12.21 with a length of 3.617 kb and the protein with a weight of 17~25 kDa, adjacent to the regulatory regions of other IL-1 members (20, 21). The *IL-37* gene contains 6 exons, and its precursor can be alternatively spliced into 5 different variants (IL-37a, IL-37b, IL-37c, IL-37d and IL-37e). However, the function of each variant is still not fully studied (6). IL-37 was reported to exist in a unique monomer/dimer equilibrium, and the monomers determine its extracellular biological effects (22). Upon binding to mast cell-derived heparin or at a high concentration, the homodimerization of IL-37 is promoted and the dimeric forms will then attenuate the anti-inflammatory action of monomers (22, 23).

IL-37b is the largest and the most extremely studied isoform of IL-37 with 218 amino acids and intact exon end in its encoding gene (24), and almost all the experimental studies reported in the literature are focused on this subtype. IL-37b contains 5 of 6 exons (exons 1, 2, 4, 5 and 6, except exon 3) of the *IL-37* gene, resulting in the most complex, complete and abundant biological functions of this isoform, while other isoforms are either nonfunctional or functionally indeterminate (25–27). The residues between D20 and E21 at the N-terminus of exon 1 in *IL-37b* gene is considered as a caspase-1 cleavage site and engenders the mature form IL-37bΔ1-20 (amino acids 21-218) (28); there exists a second cleavage site in the sequence encoded by exon 2, leading to IL-37b being cleaved into another maturation: IL-37bΔ1-45 (amino acids 46-218). The latter form is smaller but with stronger biological activity, which has been detected in the supernatant of human embryonic kidney 293 cells transfected with *IL-37* gene (29). Exons 4, 5, and 6, whereas, are predicted to encode functional proteins involved in forming the characteristic *β*-trefoil structure of IL-1 family comprising 12 *β*-strands (24). IL-37a (exons 3, 4, 5, and 6) is the only variant that contains exon 3, which encodes a catalase cleavage site and a nuclear-localization signal (30). It has been demonstrated that IL-37a exert similar anti-inflammatory effects with IL-37b (31). IL-37d (exons 1, 4, 5, and 6) shares exons 4, 5, and 6 with IL-37a as well as IL-37b, and also encodes the *β*-clover secondary structure (3, 26, 32). However, due to the lack of exon 4, the IL-37c (exons 1, 2, 5, and 6) and IL-37e (exons 1, 5, and 6) are shown to have no biological effects (3). Together, these structural differences are thought to result in only IL-37a, IL-37b and IL-37d with biological activity (33).

### 2.2 Secretion and expression patterns of IL-37

**Figure 1:**
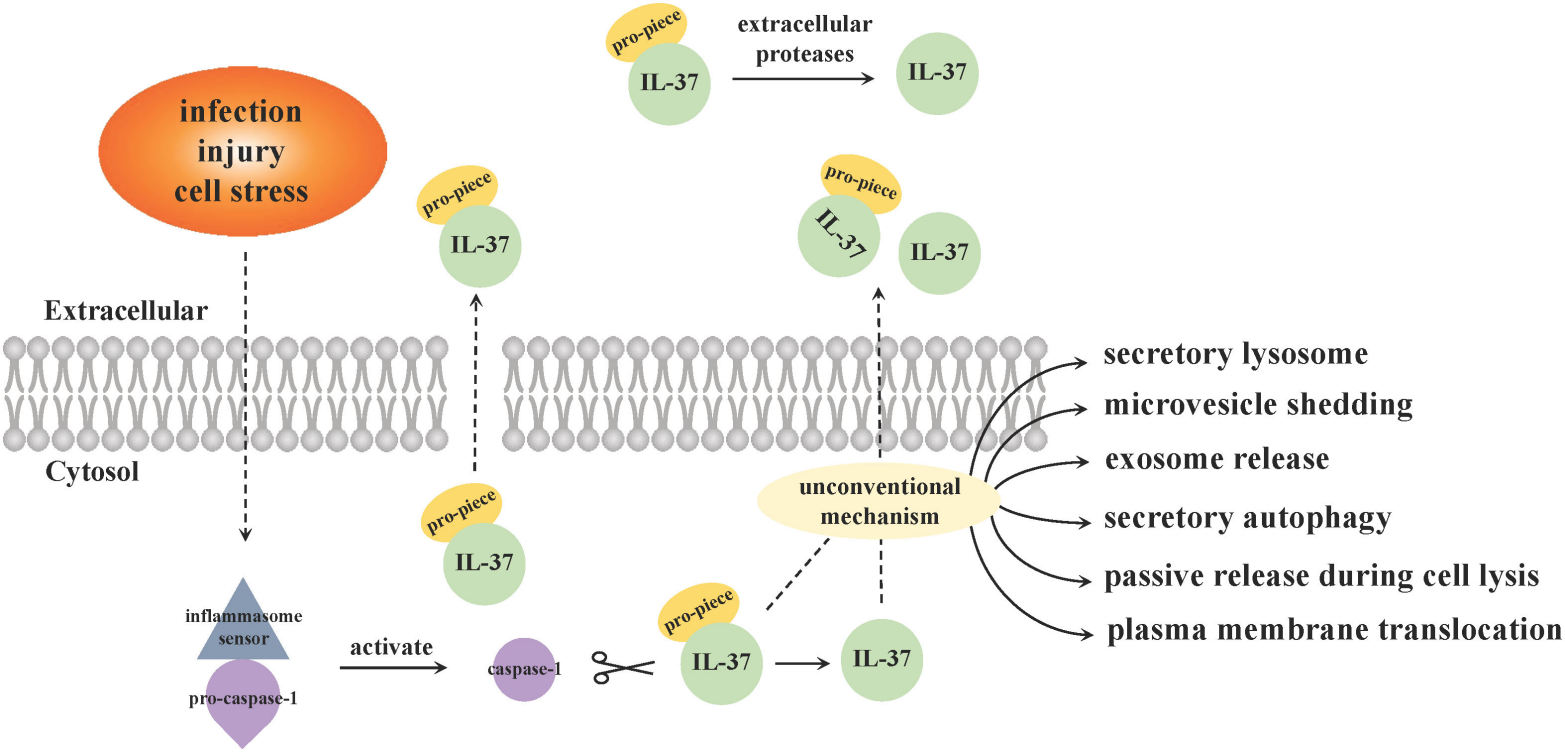
Secretion pattern of IL-37. Inflammasome sensor molecules (such as NLRP3) recognize specific danger signals, including infection, injury or cell stress, and then trigger inflammasome signal complexes assembly. Caspase-1 precursors accumulated upon the complex and are activated, which cleaves IL-37 from precursors to maturations. The secretion mechanisms of intracellular IL-37 are still not completely clear, and there are two main viewpoints. ① The precursors are released in states of cell death or lack of cell membrane integrity without caspase-1 mediation, while the cleaved IL-37 by caspase-1 intracellularly secrete from viable cells into the extracellular space via an unconventional mechanism that bypass classical ER/Golgi trafficking. ② Both precursors and mature forms of IL-37 are released from cytosol through unconventional manners. The IL-37 mature forms outside cells can also be derived from the processing of its precursors by extracellular proteases hypothesized to be secreted by macrophages.

Consistent with other members of the IL-1 cytokine family (expect for IL-1Ra), IL-37 is produced as an immature and inactive precursor with no signal peptide in the cytoplasm and nucleus, and needs to be cleaved into an active state by caspase-1 protease to become a mature form (34). Both IL-37 precursors and maturations could be detected extracellularly, while the secretory mechanisms remain unclear (**Figure 1**). Distinct from most cytokines [such as IL-10 and tumor necrosis factor (TNF)] with a signal peptide secreted via classical endoplasmic reticulum (ER)/Golgi trafficking, some scientists put forward that the precursors can be released in states of cell death or lack of cell membrane integrity in a caspase-1 activation independent manner (28). While the cleaved IL-37 that underwent an intracellular process depending primarily on caspase-1 activation are able to be secreted from viable cells into the extracellular space through an unconventional mechanism that bypass ER/Golgi secretion route, such as secretory lysosome, microvesicle shedding, exosome release, secretory autophagy, passive release during cell lysis or plasma membrane translocation (34, 35). Another study considered that IL-37 precursors could not enter into the ER as a result of lacking of signal peptide, and are released outside cytomembrane along with maturations through unconventional ways (36). The IL-37 mature forms outside cells can also be derived from the processing of its precursors by extracellular proteases hypothesized to be secreted by macrophages (26). Of note, the inflammasome [e.g., NOD-like receptor family Pyrin Domain-Containing 3 (NLRP3)] assembly is responsible for the activation of caspase-1 (28, 37), and is therefore pivotal in the maturation of IL-37. Caspase-4 can perform an analogous function with less efficiency compared to caspase-1, while other enzymes and granzymes are not involved in this process (25).

IL-37 is widely expressed constitutively in healthy human tissues, including skin, lymph node, thymus, intestine, airway, lung, bone marrow and breast, etc. (26, 38). Specifically, the five isotypes of IL-37 bear slight differences in their amino acid sequences, making them with variable tissue specific expression patterns. IL-37a, IL-37b or IL-37c is the only subtype expresses in the brain, kidney and heart, respectively, while IL-37d and IL-37e are specifically produced in bone marrow and testis (30, 39, 40). A broad spectrum of cells has been confirmed to produce IL-37 in either the cytoplasm or the nucleus, such as epithelial cells, keratinocytes, tubular epithelial cells and immune cells, including stimulated B cells, plasma cells, macrophages, natural killer cells (NKs), dendritic cells (DCs) and CD4+ regulatory T cells (Tregs) (41–43). Moreover, monocytes, purified from peripheral blood mononuclear cells (PBMCs), are the major producers of IL-37 (26, 44). Due to the half-life is limited by an instability sequence in exon 5, IL-37 mRNA is very unstable and can be easily degraded under normal conditions (45), leading to the low IL-37 protein expression and circulating concentration (<100 pg/ml) in healthy individuals (46). However, the synthesis and secretion patterns of IL-37 are altered under pathological status. A rapid upregulation of IL-37 in immune cells and peripheral circulation was observed (20) in response to inflammatory stimuli such as lipopolysaccharides (LPS) (47), Toll-like receptor (TLR) agonists, and cytokines including transforming growth factor β (TGF-β), tumor necrosis factor α (TNF-α), interferon γ (IFN-γ), as well as IL-1β or IL-18 (48, 49). As a result of the mRNA-degrading proteins being removed from the instability sequence during above inflammatory stimuli response, *IL-37* gene transcription is stabilized and IL-37 expression at both mRNA and protein levels increase (46). The expression level of IL-37 is currently thought to increase only in severe inflammatory state to avoid inflammatory intensification and immune storm, rather than in non-inflammatory or mild inflammatory conditions (47).

## 3 Molecular mechanisms of IL-37 signal transduction

**Figure 2:**
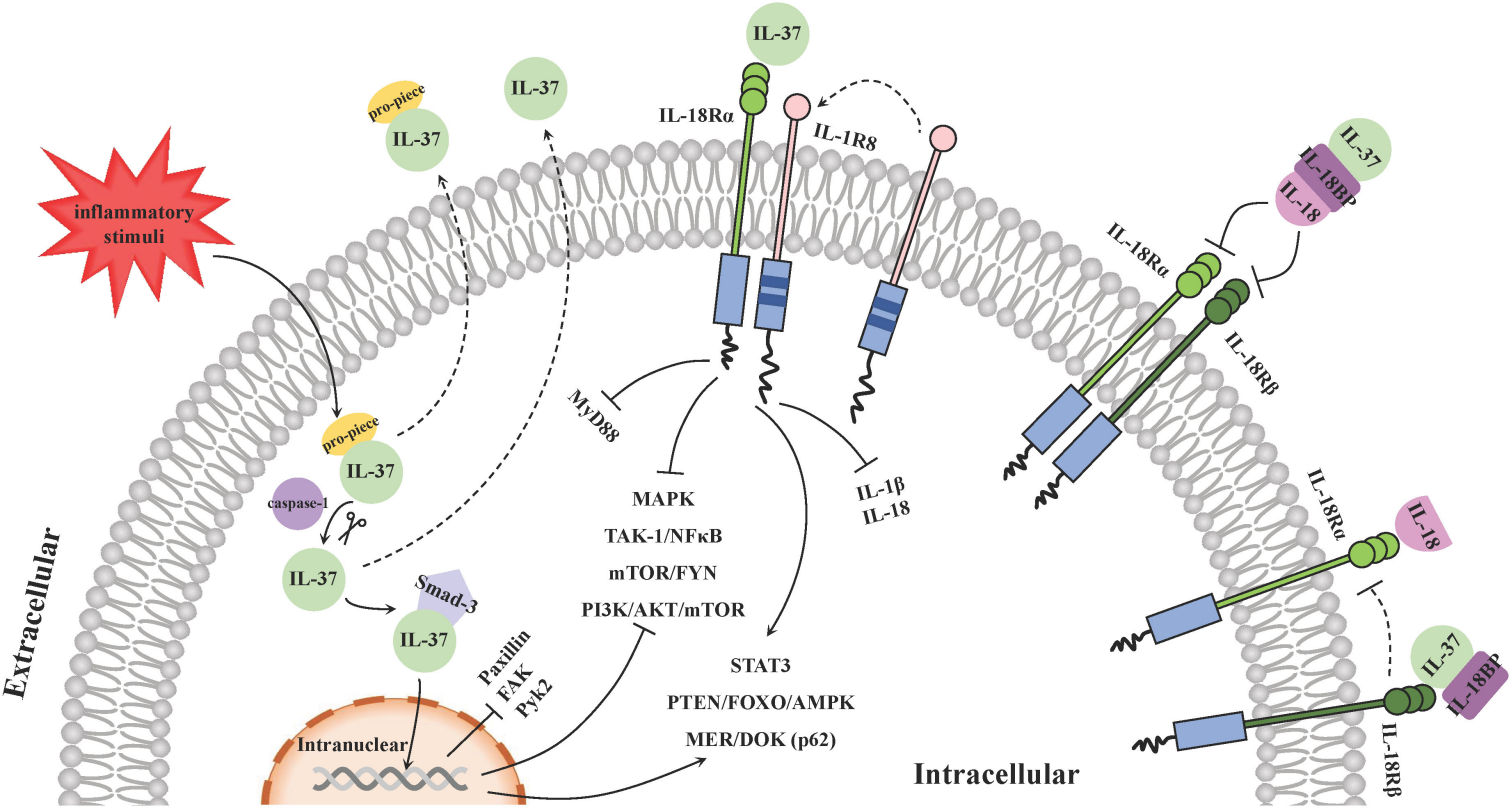
Molecular mechanisms of IL-37 signal transduction. The signal transduction of IL-37 mainly relies on intracellular and extracellular mechanisms. Inflammation stimulates the production of intracellular IL-37 precursors and then its cleaved forms by caspase-1 binds with phosphorylated Smad-3. The IL-37/Smad-3 complex traffics into the nucleus and represses the downstream inflammation-promoting kinases expression and regulates some signal pathways to exert an anti-inflammation function. Extracellularly, IL-37 binds with IL-18Rα and recruits IL-1R8 on the surface of PBMCs, such as DCs and macrophages, to form a ternary complex: IL-37/IL-18Rα/IL-1R8, and it is able to suppress pro-inflammatory cytokines and chemokines via activating and blocking various signaling pathways. In addition, IL-18BP exerts an anti-inflammatory function by binding to IL-18 extracellularly and preventing it from interacting with the receptor IL-18Rα and IL-18Rβ. IL-37 can bind to IL-18BP and enhance the function. Another ternary complex IL-37/IL-18BP/IL-18Rβ hinders recruitment of IL‐18Rβ to IL‐18Rα upon IL‐18 association, so as to counteract the pro-inflammatory effects of IL-18.

The signal transduction of IL-37 mainly relies on intracellular and extracellular mechanisms (**Figure 2**): intracellular IL-37 translocates into the nucleus in the form of a complex with Smad-3 to regulate gene expression; and extracellular IL-37 binds to surface membrane receptor IL-18 Rα and co-binding receptor IL-1R8 to activate or block downstream signaling pathways. In addition, IL-18-accociated effects are also involved in the anti-inflammatory process of IL-37.

On the one hand, inflammation stimulates the production of intracellular IL-37 precursors and triggers the activation of caspase-1, which in turn cleaves IL-37 precursors to the mature forms (20). The cleaved IL-37 (amino acids 21-218) binds with phosphorylated Smad-3 in the cytoplasm that is a kinase downstream of the TGF-β receptor (20, 50). Then the functional complex traffics into the nucleus and represses the expression of some inflammation-promoting kinases such as paxillin, focal adhesion kinase (FAK) and proline-rich tyrosine kinase 2 (Pyk2) (47). On the other hand, although no specific receptors for IL-37 have been identified (51), a large number of studies have shown that extracellular IL-37 binds with IL-18 receptor α (IL-18Rα, or IL-1R5) and recruits IL-1 receptor 8 (IL-1R8, or TIR8/SIGIRR), instead of IL-18Rβ, on the surface of PBMCs including DCs and macrophages (48), resulting the subsequent inhibition of the surrounding pro-inflammatory signal component myeloid differentiation factor88 (MyD88) signaling pathway (52). The ternary complex (IL-37/IL-18Rα/IL-1R8) promote the polarization of monocytes derived macrophages and DCs towards an anti-inflammatory or tolerate state profile via enhancing downstream STAT3 signaling pathway (53). They can also activate PTEN/FOXO/AMPK and MER/DOK(p62) (48), as well as inhibit MAPK, TAK-1/NF-κB, mTOR/FYN and PI3K/AKT/mTOR signaling activities to suppress the production of pro-inflammatory cytokines and chemokines (54–57). These inflammation inhibition regulatory pathways are also applicable to the effects of IL-37 intracellularly. In this process, the mature IL-37 binds the receptor in a more efficient manner than the precursor does (26). In addition, the production and maturation of IL-1β mediated by the assembly and activation of NLRP3 and Absent In Melanoma 2 (AIM2) inflammasome, as well as that of IL-18 mediated by NLRP3 inflammasome may also be inhibited by the ternary complex, contributing to the immune-suppressive effect of IL-37 (58, 59).

Additionally, the extracellular IL-37 is capable to bind to IL-18 binding protein (IL-18BP) and/or IL-18Rα noncompetitively (6). IL-18BP usually binds to IL-18 extracellularly with a high affinity and prevents it from interacting with the receptors IL-18Rα and IL-18Rβ, thus neutralizing IL-18 induced inflammatory activity, and the inhibitory effect of IL-18BP on IL-18 is enhanced in the presence of IL-37 (60). Furthermore, the binding of IL-37 with IL-18BP together with IL-18Rβ, forms the complex IL-37/IL-18BP/IL-18Rβ to reduce the recruitment of IL‐18Rβ to IL‐18Rα upon IL‐18 association, so as to counteract the pro-inflammatory effects of IL-18 (61). Nevertheless, it has been reported that the bind of excessive IL-18BP with IL-37 tended to reduce the anti-inflammatory activity of both IL-37 and IL-18BP (62). Recently, it was shown that different levels of IL-37 may display distinct immune-modulation capabilities. Despite that picomolar concentrations of IL-37 exerts desirable anti-inflammatory effects, for instance, it can inhibit inflammatory cytokines production including IL-6, IL-1β and TNF-α from LPS-activated PBMCs at a very low concentration (63), higher levels of IL-37 in turn weakens above effects due to its spontaneous formation of homodimers block the activity of monomers (23), which is also considered as a mechanism that automatically regulates excessive immunosuppression.

## 4 Immunoregulatory effects of IL-37

Over the past decades, the immunoregulatory function of IL-37 has been gradually clarified and proved to suppress innate and adaptive immunity. Knockdown of IL-37 in human PBMCs or macrophages increased the production of various pro-inflammatory cytokines (such as IL-1α, IL-1β, IL-6, IL-17, IL-18, TNF-α, G-CSF and GM-CSF) and chemokines (such as MIP-2/CXCL2, BLC/CXCL13, and IL-8/CXCL8) (46). It has been observed that IL-37 mutant upregulated IL-1β and IL-6 expression in PBMCs and macrophages (64), constituting a hyper-inflammatory status and inducing severe periodontal disease that displayed a striking association with oral cancer (10, 65). Mountford et al. reported that intracellular interaction of IL-37 with Smad-3 could interfere with TGF-β signaling cascade in Kupffer cells, thereby suppressing liver chronic inflammation to alleviate liver injury and fibrosis, avoiding eventual hepatocarcinogenesis (66). It has been confirmed that IL-37 could reverse obesity and insulin resistance by suppressing the phosphorylation of mTOR and activating AMPK, STAT6 and transcription factors of Foxo family (63, 67), through which IL-37 decreased inflammatory factors and chemokines derived from immune cells such as macrophages (68). Recent evidence also suggested that IL-37 reduced leukocyte recruitment by downregulating TNF-α, IL-1β and CXCL8, additionally inhibited uptake of OxLDL by IL-37-expressing bone marrow-derived macrophages, and decreased macrophage transmigration towards monocyte chemotactic protein (MCP)-1. Foam cell formation, pro-inflammatory cytokines and macrophage infiltration were reduced under these processes, making IL-37 exerting anti-atherosclerotic and plaque-stabilized maintaining effects (69, 70).

In the chronic inflammatory environment of allergic rhinitis, the interaction of IL-37 and its receptor IL-1R8 on CD4^+^ T cells were considered to negatively regulate the immune response by reducing their IL-4 and IL-17 production (71). In another airway hypersensitive state, recombined IL-37 (rIL-37) reduced airway inflammation and ameliorated asthma progression through suppressing thymic stromal lymphopoietin (TSLP) expression in lung tissues via inhibiting NF-κB and ERK1/2 pathways (72). As it has been shown that asthma was associated with increased lung cancer risk, this evidence makes IL-37 as a possible inhibitor in lung cancer (73). Furthermore, chronic low-grade inflammation or “inflammaging” is viewed as the chief culprit of impairing immunity and promoting aging, which are deemed to be associated with gradually increased incidence of cancer in the elderly (74). Recent research proved that circulating concentrations of IL-37 declined with aging. RIL-37 decreased *Pdcd1* (the gene encoding programmed cell death protein 1 [PD-1]) and increased *Lat* and *Stat4* (genes involved in T-cell activation) gene expression levels in CD4^+^T cells, as well as *Lat* in CD8^+^T cells in aged mice to young levels; besides, rIL-37 directly antagonizes TNF-α-induced programs in aging T cells. Therefore, rIL-37 treatment is believed to restore the immune function of T cells (75). IL-37 was also reported to improve hematopoiesis and B-progenitor cells functions in aged mice (75), which may represent a new strategy to overcome B-cell acute lymphoblastic leukemia (B-ALL) pathogenesis that is partly regulated by the pro-inflammatory microenvironment (76). These results provide evidence for the potential roles of IL-37 in cancer.

The IL-37/IL-1R8 axis was also speculated pivotal for restraining autoimmune response. It is believed that IL-37 prevents LPS-induced DCs from maturation and renders them tolerogenic via the IL-1R8-TLR4-NF-κB pathway during acquired immunity inhibition (77), while the tolerogenic DCs (tDCs) induce the production of antigen (Ag)-specific CD4^+^Foxp3^+^ Tregs that is conducive to building the peripheral immune tolerance and remitting inflammation (8). In addition, IL-37 can reduce the expression of the costimulatory molecules including CD40, CD80 and CD86 on the surface of DCs in the process of autoimmune response, and pro-inflammatory cytokines IL-1β, IL-6 and IL-12 were significantly decreased and anti-inflammatory cytokine IL-10 increased subsequently at both mRNA and protein levels (20). However, other studies showed that the inflammatory suppression effect of IL-37 may be independent of subsequent IL-10 expression (78). Although the increased release of IL-10 is observed mediated by human IL-37 (hIL-37) in ex vivo colonic explant tissues from dextran sodium sulfate (DSS)-induced mouse model of colitis, the protective function of IL-37 is not affected by the IL-10-receptor antibody blockade (78). Therefore, the potential mechanisms of IL-37 and IL-10 in co-regulating human immunity still need to be further explored. Moreover, the presence of IL-37 attenuated the inhibitory effect of Smad-7 on the TGF-β/Smad pathway through binding with both phosphorylated and unphosphorylated Smad-3, thereby mitigating the progress of inflammation. TGF-β/Smad pathway was viewed as a target for inflammatory bowel diseases (IBD) treatment for its roles in the suppression of DCs activation and CD8^+^ T cells function, and the promotion of tolerogenic T cells (79). In another model with DSS-induced IBD, IL-37 protein was expressed on T cells in human IL-37 transgenic (hIL-37tg) mice, and the decreased mRNA expressions of pro-inflammatory cytokines like IFN-γ, IL-1β and TNF-α in colon tissues, disease activity index (DAI) and macroscopic damage score were observed. The results of this *in vivo* study revealed IL-37-producing T cells exhibited potent anti-inflammatory and protective features in IBD (80).

## 5 Mechanisms of IL-37 in anti-cancer process

**Figure 3:**
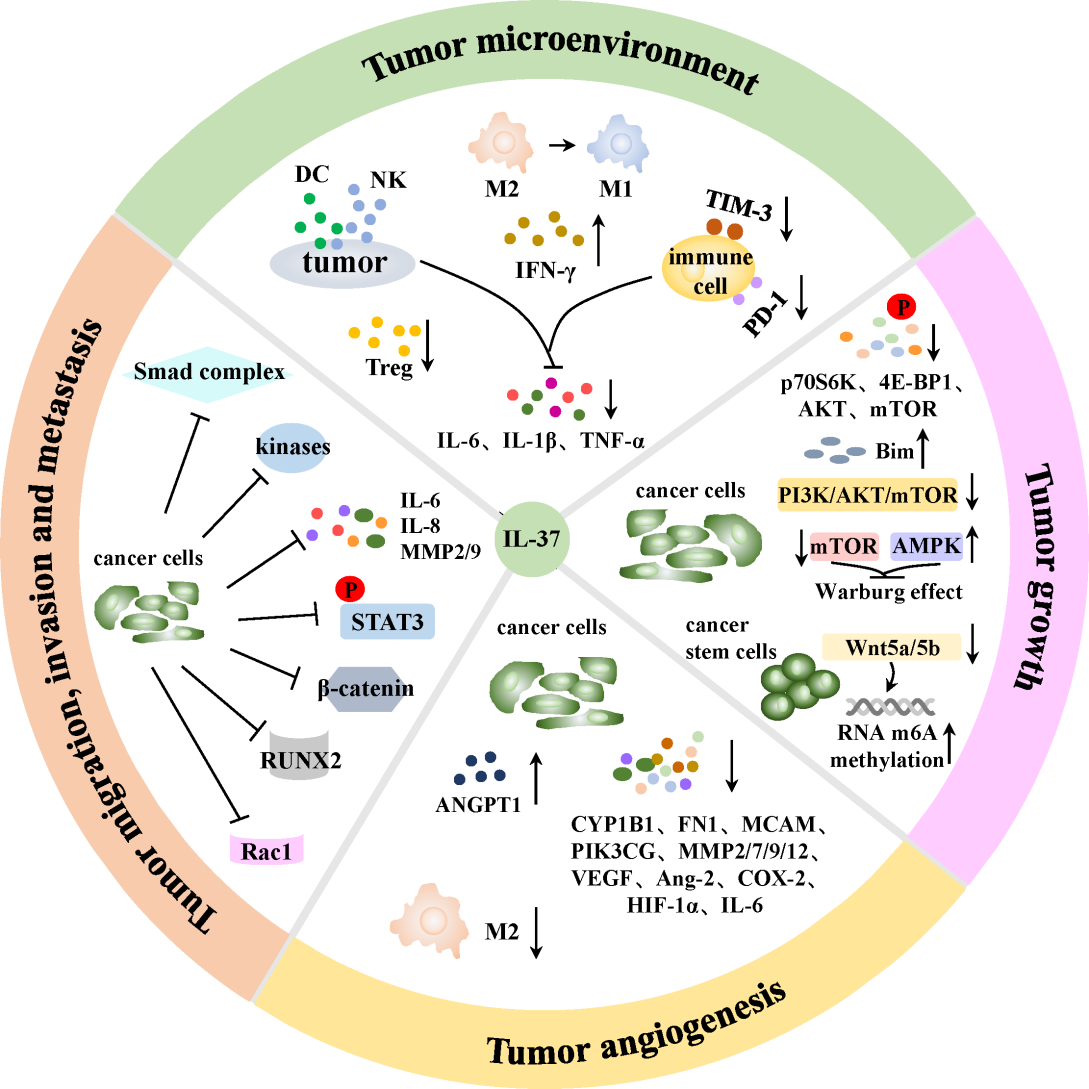
Mechanisms of IL-37 in anti-cancer process. IL-37 can stimulate DCs to release more IFN-γ to strengthen the anti-tumor activity of T lymphocytes, enhance local DCs and CD57^+^NK cells infiltration, and reduce Tregs chemotaxis in tumor sites. TAMs are able to polarize from tumor-promoting M2 to tumoricidal M1 phenotype in the presence of IL-37. Besides, PD-1 and TIM-3 immune checkpoint molecules on immune cells are induced blocked, and the production of tumor progression-associated cytokines IL-6, IL-1β and TNF-α by tumor cells together with immune cells are reduced by IL-37. Therefore, IL-37 can mediate immunoregulation in TME through above manners. Moreover, IL-37 can inhibit tumor growth via reversing the Warburg effect in cancer cells, and the suppression of Wnt5a/5b pathway indued m6A RNA methylation upregulation will inhibit the self-renewal and differentiation of cancer stem cells. IL-37 can also mediate enhanced autophagy and apoptosis of tumor cells in various ways. In addition, IL-37 can induce an antiangiogenic effect in cancer cells through increasing antiangiogenic factors expression, as well as decreasing proangiogenic factors expression and M2 TAMs maturation in tumor angiogenesis. Furthermore, IL-37 can restrain tumor cells migration, invasion and metastasis via suppressing a variety of signal pathways, kinases and cytokines in cancer cells.

Growing epidemiological evidence suggest that inflammation can increase the risk of carcinomas (38, 66, 81). Moreover, inflammatory cells and mediators present in tumor tissues are comparable to those observed in chronic inflammatory conditions (82, 83). Inflammation can induce tumorigenesis through two possible pathways: an intrinsic pathway in which chronic non-resolving inflammation (such as IBD, obesity and non-alcoholic liver steatohepatitis) increases the propensity of driving carcinogenesis, and an extrinsic pathway whereby the construction of the inflammatory microenvironment that favor tumor events (81). Given the fact that IL-37 is widely associated with inflammation, studies have shown that IL-37 was pivotal in anti-cancers processes, including immunoregulation in tumor microenvironment, inhibition of tumor growth, repression of angiogenesis and suppression of tumor cells migration, invasion and metastasis (**Figure 3**).

### 5.1 Immunoregulation in tumor microenvironment

Tumor microenvironment (TME) is typically composed of tumor cells, surrounding stromal cells (such as immune cells), extracellular matrix and signal molecules (including cytokines and chemokines) (84), in which inflammation is an essential component, constituting a hallmark of cancers (38). Chronic inflammatory condition in tumors caused by aberrant signaling following oncogenic mutations is believed to be related to the suppression of anti-tumorigenic immunity (38). As an anti-inflammatory cytokine, IL-37 is proposed to activate anti-tumor immune response by suppressing pro-tumor inflammation that releases the immune suppression in the TME (85). One possible mechanism is that the effects of IL-37 on DCs vary in different immune environments (85). It inhibits DCs function in inflammatory settings (6), while enhances DCs function in tumor models, for example, hepatocellular carcinoma (HCC) cells that overexpressing IL-37 could secrete more specific chemokines (such as CCL3 and CCL20) to increase the infiltration density of DCs in tumor sites; in addition, IL-37 could stimulate DCs to produce higher levels of cytokines such as IFN-γ, thereby indirectly strengthen the anti-tumor activity of T lymphocytes (86). Although IL-37 was reported to repress the expression of IFN-γ in IBD to reduce inflammatory response and clinical symptoms, IFN-γ protein level was also measured significantly elevated in tissues from HCC mice model after intra-tumoral injection with vaccinia virus expressing IL-37 (VV-IL-37), thereby elicited anti-tumor effect (87). Additionally, tumor cells themselves can recruit and activate specific immune cell subtypes including M2 macrophages, myeloid derived suppressor cells (MDSCs) and Tregs to create an immunosuppressive microenvironment directly (88). IL-37 is able to promote tumor-associated macrophages (TAMs) polarization from tumor-promoting M2 to tumoricidal M1 phenotype via inhibiting IL-6/STAT3 signaling as shown in cultured HCC cells (HepG2 and Huh-7) and mouse-transplanted tumor model (89); and reduce the chemotaxis of Tregs that help tumor cells escape from immunosurveillance such as in lung adenocarcinoma A549 cells (90), through which effectively promote tumor rejection. In assays in HCC Hep3B cells *in vitro* and HCC mouse model constructed of Hepa1-6 cells *in vivo*, transduced IL-37 was also verified to enhance local infiltration of CD57^+^NK cells that expresses IL-18Rα and IL-1R8 chains in tumor sites to suppress tumorigenicity (15, 91). Moreover, IL-37 reduces tumor promoting cytokines including IL-6, IL-1β and TNF-α produced by both tumor cells and immune cells (88). Furthermore, a study on aging demonstrated that IL-37 abrogated the activation of TNF-α-induced NF-κB in T cells and significantly decreased the expression of PD-1 (a classical coinhibitory factor in tumor-associated immune escape) on effector CD4^+^ and CD8^+^ T cells surface to alleviate T cells senility (75, 92). Hamilton JAG et al. also referred that IL-37 reduced the surface expression of immunosuppressive protein T cell immunoglobulin and mucin-containing molecule 3 (TIM-3) in aged backgrounds (75). TIM-3 is another emerging important immune-checkpoint molecule, whose blockade on DCs surface activated NLRP3 inflammasome and promoted puissant anti-tumor immunity (93). Recombinant IL-37 treatment in the subsequent aging B-ALL mouse model significantly strengthened anti-leukemia T-cell-mediated immune responses via preventing PD-1 expression on T cells (75). It is reasonable to suppose that IL-37 can reduce the exhaustion of immune cells in the TME and promote the anti-tumor response by downregulating the expression of PD-1 and TIM-3 protein on other immune cells surface.

### 5.2 Inhibition of tumor growth

Warburg effect refers to the aerobic glycolysis that plays an important role in the rapid growth of cancer cells. It was reported that IL-37 could reverse the Warburg effect by reducing mTOR phosphorylation and expression, together with activating AMPK pathway (94). N6-methylladenosine (m6A) is a common RNA modification that has been proved to be critical in tumorigenesis (95), as it participates in the regulation of self-renewal and differentiation of cancer stem cells (96). The overexpression of IL-37 was able to increase the overall methylation of RNA m6A via inhibiting Wnt5a/5b pathway in lung adenocarcinoma A549 cells (97), thereby suppressing tumor proliferation. Moreover, excessive autophagy is reported to be involved in the process of cell death and proliferation inhibition (98). High concentration of IL-37 mediated accelerated autophagosome formation, suppressed the phosphorylation of various proteins, including p70 ribosomal protein s6 kinase (p70S6K), 4e-binding protein 1 (4E-BP1), AKT and mTOR *in vitro* (99). Further results supplied that IL-37 induced autophagy and apoptosis by inhibiting PI3K/AKT/mTOR signaling pathway in HCC cells (SMMC-7721 and Huh-7) (99), thereby suppressing tumor growth. In addition, IL-37 was also proposed to affect autophagy through mTOR/ULK1 pathway (100). Bim is a widely recognized proapoptotic molecule of B-cell lymphoma 2 (Bcl-2) family, and acts as a crucial tumor suppressor gene taking part in the processes of apoptosis in a variety of cancers. IL-37 was reported to promote the apoptosis of cervical carcinoma HeLa and C33A cells via up-regulating the expression of Bim (101).

### 5.3 Repression of angiogenesis

A disruption of the balance between angiogenic stimulators and inhibitors may result in oncogenic angiogenesis, which has become another hallmark of cancer (11). Recent microarray studies have exhibited that IL-37 decreased the expression of proangiogenic factors such as cytochrome P450 family 1 subfamily B member 1 (CYP1B1), fibronectin 1 (FN1), matrix metalloproteinase (MMP) 2, melanoma cell adhesion molecule (MCAM) and phosphatidylinositol-4,5-bisphosphate 3-kinase catalytic subunit gamma (PIK3CG), and increased the expression of an antiangiogenic factor, angiopoietin 1 (ANGPT1) in SK-Hep-1 and SMMC-7721 cells (102), thereby exerts its tumoral angiogenesis suppression function. IL-37 could also erode angiogenesis by inhibiting another angiogenic cytokine angiopoietin-2 (Ang-2) for their serum levels were clearly negatively correlated in multiple myeloma patients (103). Interestingly, an analysis performed *in vitro* proclaimed that the direct effect of rIL-37 on human umbilical vascular endothelial cells (HUVECs) was proangiogenic, but supernatants derived from IL-37 overexpressed tumor cell lines switched its function to anti-angiogenesis. The latter indirect effect of IL-37 depended on the tilt of the balance of factors produced by tumor cells that regulated angiogenesis, which was demonstrated in murine HCC model, suggesting the dominant role of IL-37 *in vivo* was anti-angiogenesis (102). Moreover, rapid growth of tumors normally leads to a hypoxia in TME (104), in which condition TAMs are enriched (105). M2 TAMs were confirmed to be involved in tumor angiogenesis through producing proangiogenic molecules, such as vascular endothelial growth factor (VEGF), and releasing a series of enzymes that enhance angiogenesis, including MMP-2/7/9/12 and cyclooxygenase-2 (COX-2) (105). It was well-studied that IL-37 could discourage the maturation of M2 TAMs, so as to exert antiangiogenic effects (102). Various tumor cells were also reported to express angiogenesis factors under the hypoxic condition to favor tumor progression, such as IL-6, MMPs, VEGF and hypoxia-inducible factor (HIF)-1α, and IL-37 treatment was proven to prevent tumor cells from producing such factors (85, 102). Furthermore, significant decreased expressions of VEGF and MMP9 mRNA were detected in SK-Hep-1 cells transfected with IL-37 under hypoxic condition compared with those in the normal condition, indicating the antiangiogenic effect of IL-37 was amplified under the hypoxia condition in TME (102).

### 5.4 Suppression of tumor cells migration, invasion and metastasis

It is believed that both cleaved and precursor form of IL-37 could bind with Smad-3 and competitively hindered the formation of Smad-2/Smad-4/Smad-3 tripartite complex, which was responsible for the nuclear translocation of Smad-2 and Smad-4, thus inhibiting the proliferation and invasion of tumor cells in Smad signaling pathway (106). The upregulation of IL-37 may inhibit the activation of various signal phosphokinases involved in tumor metastatic behavior, including ERK 1/2, JNK, p38MAPK and PI3K (46, 106) intracellularly, and represses tumor cell migration and adhesion by downregulating paxillin, FAK and protein tyrosine kinase-2 (PTK-2) pathways (46). The expressions of potent molecules classically boost tumor cell proliferation, invasiveness and viability, such as IL-6, IL-8, MMP 2 and 9, were also verified reduced in tumor cells that dealt with IL-37 (102, 106). Furthermore, IL-37 was shown to markedly inhibit STAT3 phosphorylation dose-dependently, whose activation and expression were authenticated to increase A549 cell proliferation, invasion and oncogenic inflammation (107). Exogenous IL-37 was proved to repress the proliferation and migration of cancer cells directly through β-catenin suppression both *in vitro* (DLD1 and HT-29 cells) and in animal models of colorectal cancer (CRC) *in vivo* (108). Runt related transcription factor 2 (RUNX2) can activate genes provoking tumorigenesis and metastasis, the overexpression of IL-37 in human cervical cancer cells was validated to negatively regulate their invasion by inhibiting RUNX2 at both mRNA and protein levels (109). Additionally, the mature IL-37 transferred into the nucleus was demonstrated to restrain Rac1 activation (an important cytoskeleton modulator that regulates cell motility and was expounded to be involved in aggressive growth and malignant characteristics of HCC both in MHCC97H cells and nude mouse models (110)), and subsequently downregulated downstream PAK phosphorylation as well as epithelial-mesenchymal transition (EMT) (a biological process in which epithelial cells lose their intercellular adhesion and acquire a mesenchymal phenotype with highly migratory and invasive properties (111)), resulting in the inhibition of the angiogenesis and migration of lung adenocarcinoma cells (29).

## 6 Recent advances of IL-37 in cancer

### 6.1 Digestive system

#### 6.1.1 Hepatocellular carcinoma

HCC is a typical inflammation-related cancer caused by hepatitis B and C virus (HBV/HCV), aflatoxin exposure or alcoholism in extreme cases, the absence of active IL-37 protein may be a factor of increased risk for HCC progression in chronic HBV infected patients (16). The increased serum IL-37 levels were detected in patients with HBV infection and treated with telbivudine (16). Previous studies have revealed that Oct4 promoted HCC progression under the stimulation of IL-6 (112), and IL-37 could significantly downregulate the expression of IL-6 in the diseases. Together with the findings that Oct4 expression reduced in IL-37-transfected HCC HepG2 and MHCC97H cells, IL-37 was therefore believed to suppress HCC development through inhibiting IL-6/Oct4 pathway (113). Furthermore, IL-37 was reported to protect HCC cells against tumor progression by activating immunity in TME via regulating NK cells (15). Certain researches stated that the expression of IL-37 protein in HCC tumor tissues was decreased and negatively correlated with tumor size, while its content in surrounding healthy liver tissues was proximity to normal (15, 113). The low IL-37 expression in tumor tissues was an independent risk factor for poor prognosis and these primary HCC patients were followed for shorter overall survival (OS) and disease-free survival (DFS) (15). Thus, IL-37 may be a significant prognostic biomarker and a potential candidate as an immunotherapy avenue for HCC.

#### 6.1.2 Pancreatic cancer

Recent findings have demonstrated the role of IL-37 in pancreatic cancers, in which the pancreatic epithelial cells could undergo sustained inflammation-related adaptive response even after inflammation has subsided, accompanied with activated KRAS oncogene to promote tumor development (114). Zhao T et al. found that the levels of IL-37 protein in serum and tumor sites of pancreatic ductal adenocarcinoma (PDAC) patients were drastically lower than those of healthy controls and adjacent normal pancreatic tissues. Of note, this change of IL-37 concentrations was negatively correlated with histological grade, tumor size, lymph node metastasis and vessel invasion (115). A follow-up survey of PDAC patients revealed that patients with low IL-37 levels had significantly shorter OS and relapse-free survival (RFS) (115), indicating that the loss of IL-37 was an independent risk factor for PDAC progression. IL-37 in addition to alone was sufficient to cause a decrease in motive and migratory capacity of PDAC cells, it could sensitize and improve the efficacy of Gemcitabine that acted as the standard treatment for PDAC both *in vivo* and *in vitro* through IL-37/STAT3/HIF-1α pathway, thereby reversing the resistance in most of the treated patients and further exerting the inhibitory effect in tumor progression (115). Therefore, IL-37 could serve as a potential biomarker to evaluate disease severity, and a therapeutic strategy for PDAC.

#### 6.1.3 Colorectal cancer

Previous studies have proclaimed that IL-37 affected bile acid metabolism which has been shown to contribute to squamous cell carcinoma-associated colon cancer (116). The expression of IL-37 located in the cytoplasm of colonic epithelial cells was decreased in CRC tissues and was negatively associated with the depth of CRC invasion (70). It is believed that IBD patients with sustained intestinal epithelial inflammation are at increased risk of developing colon cancer or CRC (117, 118), while the exist of IL-37 may suppress inflammatory environment as statement above. Zhu et al. retrospectively analyzed intra-tumoral IL-37 expression in 337 CRC tissue specimens and the results showed that IL-37 deficiency was related to a poorer survival with diminished DFS and OS, suggesting IL-37 was an independent prognostic factor for CRC patients (70). Moreover, the IL-10 deficient & IL-37 transgenic (IL-10KO)/IL-37tg hybrid mouse model exhibited protective effects against subsequent inflammation and colon cancer during chronic colitis, indicating that the protective effects of IL-37 in colon cancer was associated with the regulation on IL-10 (119). Moreover, the inactivation of IL-1R8, one of the IL-37 receptors, was considered an escape mechanism of CRC (108). Thus, IL-37 may be a promising prognostic biomarker and a new feasible approach in the treatment of CRC.

#### 6.1.4 Others

Growing evidence indicates the close interwoven between IL-37 and oral squamous cell carcinoma (OSCC), possibly via counteracting the effects of IL-18 (120). It was found that serum levels and PBMC relative mRNA expression of IL-37 were significantly decreased, while those of IL-18 were obviously increased in OSCC patients (121). In addition, analysis illustrated that higher ratio of serum IL-18/IL-37 in OSCC patients was significantly correlated with shorter OS, DFS and metastasis-free survival (MFS) (121), revealing serum IL-18/IL-37 balance affected the acquired immune response and OSCC progression. Compared with OSCC patients with lymph node metastases, patients without lymph node metastases expressed higher levels of IL-37 protein (121). Furthermore, IL-37 could also inhibit the migration of OSCC via altering cell polarization (121).

Gallbladder carcinoma (GBC) is the most general malignancy of the biliary tract (122), and was verified to be associated with IL-37 functions (123). Cytological assays showed that the expression of IL-37 protein was reduced in GBC cell lines (GBC-SD and NOZ) compared with non-tumorigenic human intrahepatic biliary epithelial cell line H69. Further studies demonstrated that IL-37 inhibited HIF-1α in GBC cells and altered the expression of EMT markers dose-dependently, led to impeded the cells migration and invasion (123). It was also observed that in gallbladder cancer cells transfected with pcD-NA3-IL37, CoCl_2_ processing stabilized the expression of HIF-1α and inversely regulated the function of IL-37, GBC-SD and NOZ cells still remained a high migration capability simultaneously (123). All the evidence suggested that IL-37 could be a target for prognosis prediction and treatment of OSCC and GBC.

### 6.2 Respiratory system

#### 6.2.1 Non-small cell lung cancer

Several studies have focused on the influence of IL-37 on NSCLC, which accounts for 80-85% of lung cancer with 5-year OS rate is <15% in the US and <10% in China (107, 124). Jiang et al. clarified that IL-37 concentration in NSCLC patients’ plasma was obviously decreased, and its downregulation was closely related to the advanced Tumor Node Metastasis (TNM) stage (107). Consistently, IL-37 mRNA and protein expressions were significantly reduced in NSCLC tissues, and the descending protein was clearly associated with tumor state, TNM stage and shorter OS in patients (17). Interestingly, the expression of IL-37 genes behaved inhibiting cancer cells proliferation and advancing apoptosis only after the transplantation of a NSCLC cell line (H1299) into nude mice, but not in IL-37- transfected H1299 cell lines (17). The study also confirmed that CD34 protein levels were reduced in IL-37-overexpressing NSCLC tumor tissues, implying a decrease in microvessel density (MVD). Moreover, H1299 cells with IL-37-transfected exhibited lower VEGF levels, and IL-37 treatment apparently handicap the growth and tubule formation of HUVECs (17). These findings suggested that IL-37 may play a protective role in the development of lung cancer by inhibiting tumor angiogenesis. IL-37 may be a prognostic predictor and therapeutic target for NSCLC.

### 6.3 Reproductive system

#### 6.3.1 Breast cancer

There are few reports on the role of IL-37 in female breast cancer so far. A study utilized a mouse 4T1 breast cancer model illustrated that although IL-37 was successfully expressed in 4T1 cells after transduction with recombinant adenovirus and the secreted form was detected in the culture supernatant, the *in vitro* proliferation of 4T1 cells was not directly affected (125). In contrast, antitumor activity was found in immunocompetent BALB/c mice inoculated with 4T1-IL37 cells, and tumor growth was significantly retarded (125). This was similar to that observed in NSCLC H1299 cells (17). Further studies found that IL-37 could directly stimulate the activation and proliferation of CD4+ rather than CD8+ T cells *in vitro*, and prevent tumor growth in mice. The antitumor effects of IL-37 *in vivo* appeared to be mediated by enhancing T cell function in TME instead of affecting 4T1 cells directly (125). However, IL-37 was not regarded to obviously improve mice survival rate in the study of Wang WQ et al. (125). A recent study verified that circulating IL-37 was highest in ER+/PR+/HER2+ breast cancer patients, compared to PR+, but not ER+/PR+ patients without metastasis, suggesting that IL-37 may influence the prognosis of breast cancer via ER+/PR+/HER2+ signaling (126). Although IL-37 is protective during the development of breast cancer, there is no significant difference in IL-37 mRNA expression of patients with metastasis among ER+/PR+/HER2+, ER+/PR+ and PR+ patients, indicating that IL-37 expression may be stage-dependent, i.e. is more protective for breast cancer patients without metastasis (126). This might explain the results of Wang WQ et al. for the high metastatic characteristic of 4T1 mammary tumor cells.

#### 6.3.2 Cervical cancer

CC is the fourth most common cancer for female in both incidence and mortality, with approximately 95% cases are caused by persistent human papillomavirus (HPV) infection and related long-term chronic inflammation (127). As reported previously, IL-37 reduced the proliferation and invasion of CC cells by suppressing the expression of RUNX2 and the phosphorylation of STAT3 (109, 128). Interestingly, the expression of IL-37 mRNA was upregulated 10-fold and 2-fold in HeLa [HPV (+)] and C33A [HPV (–)] cells, respectively, after transfecting with IL-37 plasmid. This phenomenon may be associated with HPV infection, which might suggest that IL-37 may exert a higher anti-cancer efficiency in HPV (+) compared to that in HPV (–) cervical cancer cells (101). In sum, these evidences demonstrated the biological function of IL-37 and offered a potential molecule for CC treatment in the future.

#### 6.3.3 Endometrial carcinoma

Endometrial carcinoma is another most common gynecologic malignancies. Recently, IL-37 expression was demonstrated to be clearly reduced in endometrioid adenocarcinoma tissues compared to that in control endometrium and was significantly related to myometrial invasion (129). It is well-known that extracellular matrix (ECM) degradation is involved in cancer cell invasion, and MMP2 is the key molecule that degrade the basement membrane (130). Cytological experiments by Wang X et al. testified that IL-37bΔ1-45 suppressed the migration and invasion of human endometrial cancer cells *in vitro* by inhibiting Rac1/NF-κB/MMP2 signal pathway without affecting their proliferation and colony formation ability (129). In contrast to the data by Wu TJ et al. (123) and Acconcia F et al. (131), Wang X et al. found that IL-37bΔ1-45 had little effects on EMT or filamentous actin (F-actin) depolymerization of endometrial cancer cells, which were both critical to tumor cell invasion and metastasis. The tissue and cell specificity of IL-37 function may explain this discrepancy (129). The inhibitory effect of IL-37bΔ1-45 in cancer cells metastasis was also verified in a peritoneal metastatic xenograft model of endometrial cancer (129). Therefore, IL-37 might provide a novel target for the diagnosis, treatment and prognosis monitoring of endometrial cancer.

### 6.4 Urinary system

#### 6.4.1 Renal cell carcinoma

RCC is the most prevalence type of renal malignancies that derived from renal tubular epithelial cells. It has been shown that transgenic hIL-37 in tubular epithelial cells suppressed expression of IL-1β, TNFα and IL-6 induced by IL-18 in a mouse renal ischemia-reperfusion injury model, and could therefore improve mononuclear cell infiltration, kidney injury and function (132). IL-37 serum concentration in RCC patients was decreased compared to that in healthy controls, and was negatively correlated with tumor size as well as stage (19). Experiments *in vitro* verified that rhIL-37 reduced IL-6, pSTAT3Y705, cyclin D1, and HIF-1α levels that contributed to tumor cells proliferation and migration, and Bcl-2 level that inhibited apoptosis in A498 and Caki-1 cells (19). Correspondingly, following rhIL-37 administration to the induced RCC model in male severe combined immune deficiency (SCID) mice, the tumor volume and weight were significantly declined, accompanied by obvious reduced staining intensity of proliferation antigen Ki-67 and the expression of IL-6 and HIF-1α in the tumor tissue. In addition, rhIL-37-treated mice exhibited a trend toward improved survival (19). Overall, IL-37 exerts antitumor effects via inhibition of IL-6/STAT3 signaling and could be a potential agent for RCC immunotherapy.

### 6.5 Blood system

#### 6.5.1 Acute myeloid leukemia

In addition to the suppressive function in solid tumors, IL-37 was also found associated with the progression of AML, which is a hematological malignancy that generally affects the elderly (>65 years old) and is the most common acute leukemia occurs in adults (133). Previous studies have illustrated that the dysregulation of the intricate balance between pro- and anti-inflammatory cytokines in AML may affect leukemic cells proliferation, relapse and drug-resistance (134). Serum IL-37 was observed greatly downregulated in newly diagnosed AML patients, of note, IL-37 level was higher in the group with favorable prognosis than that of intermediate or poor prognosis. Furthermore, both IL-37 mRNA and protein levels restored in complete remission AML patients instead of the remained low expression in relapsed or refractory patients as the newly diagnosed ones (133). A very recent study demonstrated that AML cells secreted more pro-inflammatory cytokine IL-6 by reconstituting HS-5 cells, thereby increased the aggressive behavior of AML (135). Further analysis of the latest research found that serum IL-37 in AML patients were negatively correlated with IL-6, and rhIL-37 inhibited the expression of LPS-stimulated IL-6 in AML patients PBMCs (133), suggesting that IL-37 is involved in AML through the IL-6 signaling pathway, and may become a considerable innovative strategy for its treatment and prognosis.

#### 6.5.2 Multiple Myeloma

MM is the second most common malignant tumor of blood system (136). Highly proliferative plasma cells can induce neovascularization by releasing angiogenic cytokines under the stimulation of inflammatory factors (such as IL-6) (137); and bone marrow stromal cells secrete effective malignant plasma cell growth factors simultaneously (103). Both roles work together to promote tumor growth and resistance. Bone marrow angiogenesis drives the transition from pre-neoplastic monoclonal gammopathies of undetermined significance (MGUS) and inactive MM to active state, and its microvascular density has become a marker of MM progression and a significant prognostic factor for progression free survival (PFS) and OS in MM patients (137). A case-control study demonstrated that serum IL-37 levels in patients with active MM were significantly lower than that in healthy individuals, while serum VEGF and Ang-2 levels were obviously increased (103). Further study found that the serum IL-37 concentration decreased synchronously with the progression of MM disease, and the above two angiogenesis factors showed the opposite tendency of IL-37. In addition, rhIL-37 treatment attenuated the tube formation of HUVECs, and the secretion of VEGF in the culture supernatant was also significantly reduced (103). These results suggest that IL-37 may play an important role in angiogenesis in MM progression and provide a new idea for MM disease staging and treatment.

### 6.6 Soft tissue tumor

#### 6.6.1 Fibrosarcoma

The antitumor properties of IL-37 were first reported in 2003, when Gao et al. described that the regression of well-established intradermal MCA205 mice fibrosarcoma could be caused following single intratumor injection of constructed adenoviral vectors expressing IL-37 (AdIL-37). And complete suppression of tumor development was observed after multiple injections of the virus in most animals (18). When rechallenged at different sites with a larger dose of MCA205 cells, the researchers were surprised to find that all the tumor-mice rejected the tumor. And following multiple rechallenges, some mice rejected the injected tumor cells quickly, while those that rejected slowly eventually eradicated tumors within a short period of time (18). This manifested that AdIL-37-treated mice obtained immunity to MCA205 fibrosarcoma. However, the antitumor activity of AdIL-37 was observed abrogated in MCA205 fibrosarcoma cells-inoculated *B6.CB17-Prkdc^scid^*/*SzJ SCID* and *B6.Cg-Foxn1^nu^* nude mice that lacked functional T and B cells but with retained apparently normal Ag presentation and NK cell function. Cellular immunity may play an essential role in antitumor effects mediated by IL-37. In addition, abolished antitumor activity was also occurred in tumor-mice deficient of key cytokines in promoting antitumor cellular responses, including IFN-γ and IL-12, even though subsequent of AdIL-37 treatment. A similar result was described in a mouse model carrying homozygous mutant Fas ligand gene, where Fas-dependent pathway was predominant for IL-18 antitumor efficacy. IL-37 appeared to mediate a mixture of IL-12 and IL-18 like effects (18). On the contrary, IL-37 was capable of producing substantial antitumor effects in NKT-deficient mice (18). The data from the research explained that IL-37 exerted a significant role in the connection between innate and adaptive immunity, and may be a promising candidate for tumor immunotherapy (18).

## 7 Conclusion and future perspectives

IL-37 is a novel cytokine discovered in recent years with a broad anti-inflammation property by inhibiting inflammatory factors or molecules, regulating transcription factors and signal kinases through both intracellular and extracellular pathways in innate and acquired immunity process. With the deepening of the research on the association between inflammation and cancer, growing evidence has shown that the long-term stimulation of inflammation and the construction of inflammatory microenvironment could contribute to inducing neoplasms. Therefore, the anti-inflammatory property of IL-37 was shown to be a potential regulator in the pathogenesis of a variety of cancers. IL-37 can suppress tumor progression through multiple pathways, including mediating immunoregulation in tumor microenvironment, restraining tumor cells proliferation, controlling angiogenesis, as well as inhibiting cancer invasion and metastasis, offering valuable information for tumor immune target therapy, prognosis evaluation and medication guidance. T cells are essential for controlling the development of B-ALL, data from Hamilton JAG et al. proved that recombinant IL-37 treatment can rejuvenate aged endogenous T-cells function and boost the efficacy of aged chimeric antigen receptor T (CAR-T) cells through downregulating PD-1 surface expression (75). However, apart from the report on B-ALL, whether IL-37 can facilitate the anti-tumor response by altering immune checkpoints expression levels and their degrees including PD-1 and TIM3 in the TME of solid tumors has not been reported so far, which is worthy of further study. Perhaps IL-37 could compensate for the boundedness of immune checkpoints blockade therapy (such as PD-1/PD-L1 blockade currently applied in clinical practice but with limited effectiveness (138)), or their combined use can potentially synergize anti-tumor efficacy. Moreover, IL-37 includes five different subtypes, and almost all of the studies are aimed at IL-37b currently because of its most comprehensive and complicated biological effects. The expressions and functions of other subtypes still need further exploration. The concrete anti-inflammatory and anti-tumor mechanisms of IL-37 remains not to be thoroughly studied, warranting further detailed research on the effects of IL-37 in tumors, so as to provide new ideas for anti-cancer precise immunotherapy.

## Author contributions

MG: Investigation, Visualization, Writing – original draft. YJ: Funding acquisition, Writing – original draft. XG: Methodology, Resources, Writing – review & editing. WX: Validation, Writing – review & editing. TX: Validation, Writing – review & editing. SP: Conceptualization, Funding acquisition, Project administration, Supervision, Writing – review & editing.
